# Analytic Errors in Analysis of Public Health Survey Data Are Avoidable

**DOI:** 10.5888/pcd15.170426

**Published:** 2018-04-12

**Authors:** Brian W. Ward

**Affiliations:** 1 National Center for Health Statistics, Hyattsville, Maryland

Data from surveys are an invaluable resource for health research, and using correct statistical techniques is important when analyzing public health survey data to produce accurate findings that can inform policy and program decision-making. Yet, as a peer reviewer of scientific journals, I regularly find that many studies that analyze survey data used inappropriate methods of estimation, known as analytic error (1,2). Some examples of these errors include not applying data weights, overlooking complex survey design, and not properly subsetting data when analyzing subpopulations. Initially I found this surprising, as by the time a study is under review at a journal, multiple parties (eg, researchers, peer reviewers, journal editorial boards) have had an opportunity to identify these analytic errors.

Consistent with my own experience as a peer reviewer, empirical studies have found this type of error to be common (1,2). A meta-analysis of 100 peer-reviewed journal articles that performed secondary analysis of data from nationally representative surveys with complex sample designs found 1,100 instances in which analytic errors may have occurred, and in 616 instances these errors were likely present (1). Of the articles reviewed, 28% did not mention weighting data, 40% did not mention accounting for complex sample design to properly estimate variance, 59% used improper language when discussing results in the text (eg, estimates generalized to the “sample” as opposed to the “population”), and 79% did not use proper significance testing (1). These results are particularly alarming because the meta-analysis reviewed articles *published* in peer-reviewed journals.

## Empirical Example

Although previous work has documented the extent of analytic errors in published studies that describe analysis of data from surveys with complex design (1,2), understanding how committing such errors could affect one’s empirical findings, and the conclusions drawn from them, is important. To show this, I used data from the 2013 National Ambulatory Medical Care Survey (NAMCS) and provide the following example that illustrates the consequences of committing analytic errors, specifically not weighting or accounting for sample design when analyzing data from a nationally representative survey (Table). This example examines the percentage difference in physician office visits made by non-Hispanic white and Hispanic adults with multiple chronic conditions (MCC).

**Table:** Percentage of Physician Office Visits by Non-Hispanic White and Hispanic Adults With Multiple Chronic Conditions, National Ambulatory Medical Care Survey, 2013^a^

Statistical Test	Series 1: Data Unweighted, Complex Sample Design of Study Unaccounted For	Series 2: Data Weighted, Complex Sample Design of Study Unaccounted For	Series 3: Data Weighted, Complex Sample Design of Study Accounted For
Non-Hispanic white (*X_1_*), % (95% CI) [SE]	34.3 (33.80–34.78) [0.25]	35.8 (35.11–36.56) [0.37]	35.8 (34.21–37.49) [0.84]
Hispanic (*X_2_*), % (95% CI) [SE]	30.3 (28.76–31.80) [0.78]	33.0 (30.80–35.31) [1.15]	33.0 (28.64–37.71) [2.32]
*X_1_*-*X_2_*	4.0	2.8	2.8
Significance test statistic	4.88	2.32	1.13
*P* value (2-tailed)	<.001	.02	.26

Abbreviations: CI, confidence interval; SE, standard error.

a Unweighted sample size includes 12,216 visits by non-Hispanic white adults and 1,063 visits by Hispanic adults. All estimates produced using SAS-callable SUDAAN (version 11.0) with proper subsetting procedures. Multiple chronic conditions defined as having been diagnosed with ≥2 of 13 conditions: arthritis, asthma (current), cancer, cerebrovascular disease (includes stroke), chronic obstructive pulmonary disease, chronic renal failure, congestive heart failure, depression, diabetes, hyperlipidemia, hypertension, ischemic heart disease, and osteoporosis (3). A description of the data (including response rate) can be found at ftp://ftp.cdc.gov/pub/Health_Statistics/NCHS/Dataset_Documentation/NAMCS/doc2013.pdf.

In the first series of estimates, I considered neither weights nor NAMCS sample design. The result shows that the percentage of office visits among non-Hispanic white adults with MCC was significantly higher (*P* < .001) than that of Hispanic adults with MCC (34.3% vs 30.3%, respectively). Without weighting, these estimates are only for the 13,279 visits sampled; therefore, using the estimates to generalize to office visits for all US adults would be inaccurate.

In the second series, I applied weights without accounting for the NAMCS sample design. The percentages change to 35.8% for non-Hispanic white adults and 33.0% for Hispanic adults. The difference still appears significant (*P* = .02) but only by 2.8 percentage points in magnitude, less than the 4.0 percentage-point difference between the unweighted estimates. Although by using weights these estimates are now generalizable to all physician office visits by US adults, and no longer limited to only the 13,279 visits included in the sample, this difference between the estimates is invalid without accounting for the complex sample design of NAMCS.

In the third series, I used the proper procedure by weighting the data and accounting for the survey’s sample design. The percentages remain unchanged; however, using sample design information properly accounts for additional covariance attributed to clustering from the 2-stage, stratified design of the 2013 NAMCS. This is evident by wider confidence intervals and larger standard errors in the third series of estimates, compared with those in the first and second series. As a result, the difference between the 2 racial/ethnic groups is no longer significant (*P* = .26).

In this example, not properly accounting for sample weights and complex sample design would have led to inaccurate estimates not generalizable to the target population and erroneous reporting of a significant difference that does not exist (type I error). More resources detailing how to properly analyze public health survey data (eg, Korn and Graubard’s widely used *Analysis of Health Surveys*) are available (4).

## Shared Responsibility

Many have argued that prevention of analytic errors lies primarily with the researcher, who must understand the survey data, use appropriate estimation techniques when analyzing them, use proper language when describing methods and results, and ideally have a coauthor or colleague verify estimates and proper use of the software programs used to generate the estimates (5). In fact, with proper training and understanding, researchers who analyze survey data should be able to remove this source of error from their research. However, other parties have a role as well (1,2), and actions are being taken to help prevent and remove analytic error from empirical studies.

For example, the National Center for Health Statistics — the organization that collects and releases NAMCS and other public health survey data — ensures that detailed documentation on each survey is accessible and that it clearly describes how to properly analyze the data. The peer-review process is important to identify analytic errors and preclude manuscripts with these errors from being published. This review process involves both peer reviewers as experts in the field and editors of the journal, who both check for these types of errors. If it is unclear whether an analytic error is present, the reviewer can notify the author and editors and request for clarification. If a reviewer is not familiar with the appropriate estimation techniques, the editor in chief will seek statistical consultation from another reviewer. Some journals’ editorial boards provide guidance for reporting analyses of survey data, and many ensure that statistical reviewers assess certain manuscripts (eg, *Preventing Chronic Disease* for example, does this). Having statistical reviewers is associated with lower prevalence of analytic errors (1). Finally, professional organizations such as the American Association for Public Opinion Research have developed guidelines (or “Best Practices”) on analyzing and reporting survey data using appropriate methods of estimation, similar to the approach of guidelines that are present for randomized, controlled trials (ie, Consolidated Standards of Reporting Trials [CONSORT] statement [6]) and qualitative research (ie, Consolidated Criteria for Reporting Qualitative Studies [COREQ] checklist [7]).

Analytic errors are avoidable, yet if unchecked they can have adverse consequences to our understanding of various health topics and the potential to misguide future research. Although the responsibility of their prevention primarily belongs to the researcher, other parties such as organizations conducting surveys, peer reviewers, journal editors, and professional associations share this responsibility. Making a collective effort to reduce analytic errors in health survey research is important for generating accurate results and making better-informed policy and programmatic decisions.
